# ncBAF recognizes the nucleosome through BCL7A in chromatin remodeling

**DOI:** 10.1038/s41421-025-00858-1

**Published:** 2025-12-16

**Authors:** Kangjing Chen, Liwen Du, Yumin Liu, Mo Chen, Zhucheng Chen

**Affiliations:** 1 Key Laboratory for Protein Sciences of Ministry of Education, School of Life Science, Tsinghua University, Beijing, China; 2 Beijing Advanced Innovation Center for Structural Biology & Beijing Frontier Research Center for Biological Structure, School of Life Science, Tsinghua University, Beijing, China; 3 Tsinghua-Peking Center for Life Sciences, Beijing, China; 4 SXMU-Tsinghua Collaborative Innovation Center for Frontier Medicine, Shanxi Medical University, Taiyuan, Shanxi China; 5 State Key Laboratory of Molecular Oncology, School of Basic Medical Sciences, Tsinghua University, Beijing, China

**Keywords:** Cryoelectron microscopy, Chromatin remodelling

Dear Editor,

The SWI/SNF family complexes are highly conserved across evolution, and play a critical role in regulation of gene expression. The SWI/SNF homologs in mammalian cells (mSWI/SNF) are often referred to as the Brg1-asscoiated factor (BAF) complexes. In line with their broad functionality, mutations of the BAF complexes are found in over 20% of human cancers^1^, whereas accumulating evidence indicates that the intact complexes are required for tumorigenesis of many cancer types^2^. The BAF complexes are assembled into three major forms: canonical BAF (cBAF), Poly-bromo associated BAF (PBAF), and non-canonical BAF (ncBAF). Different from cBAF and PBAF, ncBAF lacks the essential nucleosome-binding subunit SMARCB1 and the scaffold subunit (ARID1A in cBAF or ARID2 in PBAF)^3^. While the structures of cBAF and PBAF bound to the nucleosome were determined^4–7^, how the ncBAF complex interacts with the nucleosome substrate remains unknown. Here we report the cryo-electron microscopy (cryo-EM) structure of the human ncBAF complex bound to the nucleosome, revealing the unique mechanism of nucleosome recognition by ncBAF.

Previous studies indicate that the SWI/SNF family complexes are generally delineated into three functional modules: the motor module, the actin-related protein (ARP) module, and the substrate recruitment module (SRM)^2,8^. ACTB and ARP4 are organized by the HSA-helix of the motor to form the ARP module, while the other auxiliary subunits are assembled into the SRM. The human ncBAF complex was reconstituted with the motor subunit SMARCA4 and 8 auxiliary subunits, including SMARCC1, GLTSCR1L, SMARCD1, BRD9, SS18-SSX, ARP4, ACTB and BCL7A. The purified complex was mixed with nucleosome core particles (NCPs) in the presence of stable ATP analog (ADP-beryllium fluoride, BeF_x_), and analyzed by cryo-EM (Supplementary Fig. S1). The structure of the ncBAF complex bound to the nucleosome was determined at an overall resolution of 4.0 Å, which reveals the envelope of the holo-complex (Fig. 1a). The local maps around nucleosome-motor-ARP, the nucleosome and the ARP module were refined to 2.9 Å, 2.4 Å and 3.4 Å, respectively (Fig. 1b; Supplementary Fig. S1 and Table S1). Whereas the structures of the motor and ARP module are well determined, the structure of SRM was not resolved in the current study. AlphaFold3 predicts an elongate-shaped SRM, which is temporally fit into a weak EM density of the overall map at a lower contour level (Supplementary Fig. S2a), suggesting that SRM in ncBAF is highly flexible.

**Fig. 1:**
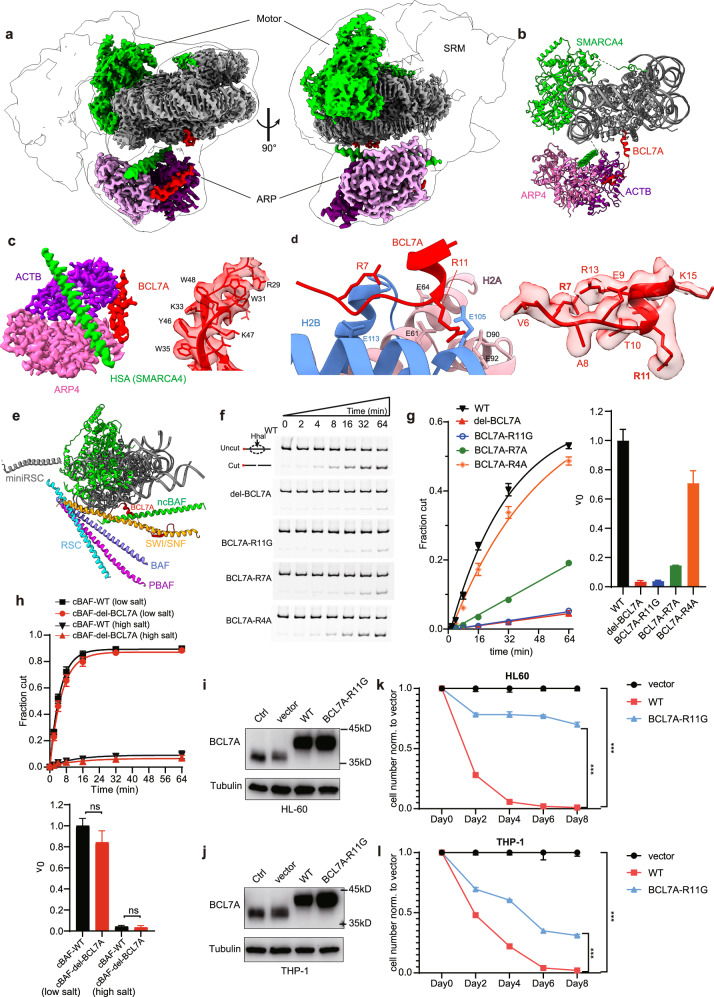
Structure of the ncBAF complex bound to the nucleosome and the importance of BCL7A in chromatin remodeling. **a** Two different views of the composite cryo-EM map of the ncBAF–NCP complex, with the molecular envelope outlined. **b** The ribbon model of ncBAF–NCP complex. **c** Local EM map of the ARP module. The bound BCL7A fragment is shown on the right. **d** Binding of the RA motif of BCL7A to the AP of H2A-H2B. The local EM density map of the RA motif is shown on the right. **e** Different conformations of HSA-helix in the SWI/SNF family complexes. The models (cBAF: 6LTJ^4^, PBAF: 7VDV^6^, SWI/SNF: 7EGP^8^, RSC: 6KW3^14^ and miniRSC: 6VZ4^15^) are aligned according to the nucleosome, and only HSA domains and BCL7A are shown for clarity. **f** Representative gels of the chromatin remodeling activities of WT and BCL7A mutant ncBAF at the physiological salt condition (150 mM NaCl). **g** Quantification of the chromatin remodeling activities of WT (black) and BCL7A deletion (red) or R11G (blue), R7A (green), R4A (orange) mutant complexes. Normalized initial remodeling rates (v_0_) are shown on the right. Data are mean ± SD (*n* = 3 technical replicates). **h** Quantification of the chromatin remodeling activities of WT (black) and BCL7A deletion (red) cBAF. Low salt, 100 mM NaCl; High salt, 150 mM NaCl. Data are mean ± SD (*n* = 3 technical replicates). Representative gels are shown in Supplementary Fig. S4c. **i**, **j** Western blot analyses of the overexpression of WT and mutant BCL7A in HL-60 and THP-1 cells. **k**, **l** Cell growth assays showing that overexpression of BCL7A-WT significantly inhibited the proliferation of HL-60 and THP-1 cells, whereas the R11G mutant partially attenuated this inhibitory effect.

The high resolution map of the ARP module reveals the previously unresolved subunit BCL7A, which interacts with ACTB and ARP4 via a two-stranded β-sheet (residues 28–51), instead of direct interaction with the HSA helix as proposed before (Fig. 1c)^9^. The structural models were built by fitting available structures^6^ and predicted structures by Alphafold3 into the cryo-EM maps, followed by manual model building, confirmed by cross-linking mass spectrometry (XL-MS) (Supplementary Fig. S2a, b). Within the SRM, Alphafold3 predicts with high confidence (PAE < 5) that the ncBAF specific subunit GLTSCR1L interacts with coiled-coil SMARCC1 and SMARCD1 subunits mainly through a long helix, the interactions of which are confirmed by XL-MS (Supplementary Fig. S2c, d). The SRM of ncBAF is different from those of cBAF and PBAF, which are organized by multiple armadillo repeats of ARID1A and ARID2, and well positioned at the side of the nucleosome^4,6^. The structural plasticity of SRM of ncBAF is probably an inherent property of the complex, which lacks a rigid scaffold subunit.

The ARP module of ncBAF adopts a conformation notably different from those in other SWI/SNF family complexes (Fig. 1e). The HSA-helix of ncBAF is in close proximity to the nucleosome, running in parallel to the disc-surface, whereas the HSA-helixes in other SWI/SNF family complexes tilt to different angles. The HSA-helix and associated ARPs are known to modulate the motor activity of Snf2-like enzymes^2^, we speculate that the distinct conformations of the ARP module in human BAF complexes could contribute to different regulation of the SMARCA4 motor.

Importantly, the N-terminal fragment of BCL7A in ncBAF binds to the H2A-H2B acidic patch (AP) of the nucleosome (Fig. 1d). Residues 6–10 of BCL7A adopt a loop conformation and residues 11–15 form a short helix, with Arg11 and Arg7 anchored at the canonical and variant AP pockets, respectively^10^. In cBAF/PBAF, SMARCB1 is bound to the AP of the nucleosome^2,4,6^. ncBAF lacks SMARCB1, and the arginine anchor motif of BCL7A plays a similar role in nucleosome recognition (Supplementary Fig. S3a). Both BCL7A in human ncBAF and Rtt102 in the yeast SWI/SNF interact similarly with ARPs (Supplementary Fig. S3d, e), despite with low sequence homology (15.9% identity, Supplementary Fig. S3f). The arginine-anchor motif is present in BCL7A but not in Rtt102, suggesting that this interaction represents a distinct mechanism of nucleosome recognition in the human complexes.

To evaluate the importance of nucleosome binding by BCL7A, we deleted the BCL7A subunit, and mutated the canonical arginine anchor (R11G), which is recurrently found in diffuse large B-cell lymphoma (DLBCL) cancer patients^11^. The mutation or deletion of BCL7A did not perturb the assembly of the ncBAF or cBAF complexes (Supplementary Fig. S4a, b). Relative to the wild-type (WT) ncBAF complex, the R11G mutant and BCL7A deletion displayed over 10-fold lower remodeling activities (Fig. 1f, g). At a low salt condition, the complexes exhibited higher activities, and both mutants displayed over 3-fold reduction of remodeling activity (Supplementary Fig. S4d, e). These findings indicate that nucleosome recognition by BCL7A is critical for the remodeling activity of ncBAF. In contrast, BCL7A appears less critical for cBAF, as there was no statistically significant difference in chromatin remodeling between the WT and BCL7A-mutant cBAF complexes under any tested conditions (Fig. 1h; Supplementary Fig. S4c). The different sensitivity to the loss of BCL7A is in line with the different modes of nucleosome engagement by cBAF and ncBAF.

The AP recognition mode of BCL7A observed in study is different from the structure of isolated BCL7A bound to the nucleosome determined previously, in which Arg4 recognizes the canonical AP pocket (Supplementary Fig. S3b, c)^12^. To further test the functional relevance of the structures, we compared the remodeling activities of the R7A and R4A mutant ncBAF. The R7A mutant showed a ~ 6-fold reduction in the remodeling activity, whereas only a slight reduction was observed in the R4A mutant (Fig. 1g). Together with the biochemical analyses above, the data support our structural model that Arg11 plays a major role by recognition of the canonical AP pocket and Arg7 binds to the adjacent variant site, while Arg4 is in less important in context of the ncBAF complex.

To investigate the importance of Arg11 in vivo, we stably expressed WT and mutant BCL7A into two acute myeloid leukemia (AML) cell lines, HL-60 and THP-1, in which BCL7A functions as a tumor suppressor^13^. Western blot analysis confirmed the robust expression of both WT and R11G mutant BCL7A (Fig. 1i, j), demonstrating that the exogenous proteins were effectively synthesized and stably expressed in these cell lines. The cell proliferation assays provided a compelling demonstration of BCL7A’s regulatory influence on cell growth (Fig. 1k, l), consistent with the previous studies^13^. In contrast, the R11G mutant largely released the inhibition effect (Fig. 1i–l), supporting that Arg11 is crucial for BCL7A’s tumor suppressor activity.

Taken the structural, biochemical and cellular data together, our studies indicate an unexpected nucleosome binding mode of ncBAF, and provide mechanistic insights into the importance of BCL7A as a tumor suppressor. Specifically, our findings highlight the critical role of BCL7A of the ncBAF complex in nucleosome recognition and chromatin remodeling. This functional requirement is particularly demonstrated in our AML cell models, where the R11G mutation in BCL7A substantially compromises its tumor suppressive activity. These discoveries not only enhance our understanding of the functions of the SWI/SNF family complexes but also provide potential therapeutic targets for the development of strategies against related cancers.

## Supplementary information

Supplementary Information

Supplementary Table S2

## Data Availability

Coordinates and EM maps have been deposited in the EMData Resource and PDB under accession codes EMD-65852 (ncBAF-NCP), EMD-65851 (NCP-motor-ARP) with related PBD 9WBZ, EMD-65853 (NCP-RA) with related PBD 9WC0, and EMD-65854 (ARP) with related PBD 9WC1.
