# Supplementary material for: ncBAF recognizes the nucleosome through BCL7A in chromatin remodeling
Source: Cell Discov. 2025 Dec 16;11:102. doi: 10.1038/s41421-025-00858-1 (PMC12708618; doi:10.1038/s41421-025-00858-1)
Supplement: Supplementary file 1 — Supplementary Information [file 41421_2025_858_MOESM1_ESM.pdf]

Materials and Methods

Expression and purification of ncBAF

The nine ncBAF subunits were sub-cloned into pCAG vectors. To facilitate protein purification, his-tag was added to the C-terminus of SMARCD1, and strep-tag was added to C-terminus of GLTSCR1L. To improve protein expression, we removed the internal non-conserved region of GLTSCR1L (residues 520-620) at first (ncBAF-Nucleosome dataset1), which is not required for the complex integrity. ncBAF with full-length GLTSCR1L was used in dataset2. HEK293F cells were grown in SMM 293-TII medium (Sino Biological Inc.) to a density of ~2.5×10^6 cells per ml. The plasmids were transiently transfected to HEK293F cells using polyethyleneimines (PEI) (Yeasen Biotechnology). Plasmids (2.4 mg in total) and 9 mg PEI were mixed in 80 ml fresh medium for 20–30 min, then added to the 800 ml culture. The transfected cells were cultured at 37 °C supplemented with 5% CO_2 in a shaker for 72 h. Cells were harvested by centrifugation and resuspended in cold lysis buffer containing 50 mM Tris, 300 mM NaCl, 2 mM $MgCl_2$, 10 mM imidazole and protease inhibitor cocktails (5 μg ml^{-1} aprotinin, 5 μg ml^{-1} leupeptin and 2.5 μg ml^{-1} pepstatin), pH 7.5. Cells were lysed by ultrasonication and cell debris were removed by centrifugation at 19,000 rpm at 4 °C for 1 h. The supernatant was collected and applied to a 5 mL HisTrap HP column (Cytiva). The column was washed with 25 column volumes high salt buffer containing 50 mM Tris, 500 mM NaCl, 2 mM $MgCl_2$, 20 mM imidazole and protease inhibitor cocktails, pH 7.5, followed by 5 column volumes low salt buffer containing 50 mM Tris, 150 mM NaCl, 2 mM $MgCl_2$,

20 mM imidazole and protease inhibitor cocktails, pH 8.0. The bound proteins were eluted with elution buffer containing 50 mM Tris, 150 mM NaCl, 2 mM $MgCl_2$, 300 mM imidazole and protease inhibitor cocktails, pH 8.0, and then applied to a StrepTrap HP column (Cytiva) and washed with 30 column volumes strep-wash buffer containing 50 mM Tris, 150 mM NaCl, 1 mM EDTA, pH 8.0. Proteins were eluted with strep-elution buffer (strep-wash buffer supplemented by 8 mM D-desthiobiotin). The sample was then applied to Mono Q column (Cytiva), washed and eluted with a linear gradient from 0–100% QB-buffer containing 50 mM Tris, 1 M NaCl, 5% glycerol, 2 mM DTT, pH 8.0. ncBAF-containing fractions were collected and concentrated to around 1 mg ml^{-1}. The mutant proteins were expressed and purified following the same procedure. cBAF was prepared similarly using the full-length SMARCC1 homo-dimer[1]. All other 10 subunits are full length proteins, except the residues (1-990) of ARID1A was truncated. N-terminal strep-tag, and C-terminal his-tag were added to ARID1A and SMARCD1, respectively, to facilitate protein purification.

Preparation of nucleosomes

Mono-nucleosome was reconstituted with Xenopus histones and DNA containing Widom 601 positioning sequence with flanking linker DNA 20 bp and 40 bp at either ends (20N40) as the substrate for the determination of the structure of ncBAF bound to the nucleosome. The 100N100 nucleosome was used as the substrate in the nucleosome remodeling assays. The specific sequences of the DNA (20N40 or

100N100) were the same as published before[2]. The 20N40 nucleosome with short linker DNA could be reconstituted with higher yield and purity, and less free DNA, which are good for cryo-EM analysis. The Hha1 cutting site is buried inside the nucleosome, and fully exposed after translocation over ~70 bp. So, the 100N100 nucleosome with long linker DNA were used for the cutting assay to increase the cutting efficiency.

Cryo-EM sample preparation

The ncBAF complexes were mixed with the 20N40 NCP at a ratio of 1:2 in the presence of ADP-BeFx in a similar way as described before[2]. After dialysis for 3-4 hours, the complex was subjected to Grafix treatment (0-0.15% glutaraldehyde). The peak fraction was concentrated for electron microscopy analysis. Negative staining of the ncBAF-nucleosome complex was performed with 3% uranyl acetate. Grids were examined using an FEI T12 microscope operated at 120 kV to verify the appropriate concentration for the cryo-EM sample analyses.

To prepare cryo-grids, Quantifoil gold R2/1 grids with 200 mesh size were subjected to glow discharge in air for 30 s using a PDC-32G-2 Plasma Cleaner set to a low power. Samples (4 μL at 1μM) were blotted for 3.5 s at -2 force before being plunge-frozen in liquid ethane with a FEI Vitrobot IV at 8 °C and 100% humidity.

Grids were examined and screened using a FEI Tecnai or Talos Arctica operated at 200 kV. Cryo-EM data were collected using a Krios G3i (Thermo Fisher) operated at 300 kV equipped with a K3 direct electron detector and GIF Quantum energy filter (Gatan), at a nominal magnification of 81,000× for a final pixel size of 0.5412 Å/pixel with the defocus values ranging from -1.4 to -1.8 μm. The total electron dose was 50 $e^{-}/Å^2$ fractionated in 32 frames (exposure time 2.56s). AutoEMation II (developed by Jianlin Lei) was used for automated data collection.

Image processing

We collected 9,143 micrographs of the ncBAF–Nucleosome sample with GLTSCR1L-truncation (dataset1, Supplementary Fig. S1a), and 3,349 micrographs of the GLTSCR1L-FL (dataset2, Supplementary Fig. S1b). All these image stacks were aligned using MotionCor2[3] with twofold binning, and CTF parameters were estimated using CTFFIND4[4]. Particle picking, two-dimensional (2D) classification and three-dimensional (3D) classification were carried out in Relion3.0[5] or Cryosparc4.4[6]. The initial 3D model of the ncBAF-nucleosome complex was constructed using Cryosparc4.4. The initial picked particles (5,534,666 from ncBAF–Nucleosome dataset1 and 1,526,709 from ncBAF–Nucleosome dataset2) were extracted with fourfold binning to increase signal to noise ratio. Owing to the high similarity, the two datasets were combined and multiple rounds of 3D classification or heterogenous refinement were performed to remove broken particles. All the 2,300,342 particles with clear NCP-motor-ARP envelope were combined and performed heterogenous

refinement in Cryosparc4.4 to purify particles with clear motor and ARP bound to the nucleosome. The best class with 175,820 particles with clear NCP-motor-ARP density was selected and refined to 2.9 Å. All the 635,115 particles with complete ARP were re-extracted without binning and local refined with a mask on NCP-RA region, and reference based motion correction and global CTF refinement were performed in Cryosparc4.4 to get the structure of NCP-RA at a resolution of 2.4 Å. Global 3D classification in Relion3.0 was performed to purify the best 106,020 particles with SRM envelope and after re-extraction these particles without binning, we got the overall structure of ncBAF-Nucleosome at a resolution of 4.0 Å. To further improve ARP module, local search in 3D classification with a mask on ARP was performed in Relion3.0. The best 55,992 particles with clear secondary structures were local refined with a soft mask on ARP module and yield a resolution of 3.4 Å.

Model building

The initial model was built by fitting the maps in Chimera[7] using the known structure of SMARCA4-nucleosome complex (PDB code 7VDT)[2] and the ARP module predicted by Alphafold-multimer[8] as the templates. The SRM module was predicted using Alphafold3[9] and the loops with low confidence were deleted manually. The atomic model for the BCL7A was built manually in Coot[10]. The structures were refined using Phenix[11] with secondary structure constrains. The map and model representations in the figures were prepared by UCSF Chimera or UCSF ChimeraX[12]. The overall structure of ncBAF-nucleosome in Fig. 1a was presented using a low-pass

filter to the map using Chimera's Volume Filter tool to achieve smoother structural features.

Nucleosome remodeling assay

Restriction enzyme accessibility assays were performed as described[2]. To compare the remodeling activities of ncBAF, 5 nM protein complex was incubated with 10 nM Cy5-labelled 100N100 nucleosome at 30 °C in the remodeling buffer containing 3 mM ATP and 1 U μl^{-1} HhaI, 20 mM Tris, 150 mM NaCl, 5% glycerol, 5 mM $MgCl_2$, 0.1 mg ml^{-1} BSA, pH 8.0. The cBAF has a very low remodeling activity under 150 mM NaCl, and a lower salt concentration (100 mM NaCl) was also used. The reactions were quenched with 2× Stop buffer containing 20 mM Tris, 1.2% sodium dodecyl sulphate (SDS), 80 mM EDTA, 0.2 mg ml^{-1} proteinase K, pH 8.0, and further incubated at 55 °C for 20 min to deproteinate the samples. Samples were loaded on 8% (37.5:1) native polyacrylamide gel and run in 0.5×TBE for 60 min at 150 V. Gels were scanned using Typhoon FLA9500 imager and band intensity was quantified by Quantity One software. The data with high remodeling activities were fitted to one phase decay equations using GraphPad Prism. The initial rates were calculated by taking the derivative of each exponential fit at time zero.

XL–MS analyses

The XL–MS analyses were performed as previously described[2]. The purified full-length ncBAF complex (1 μM) was preincubated with 20N40 nucleosome (2 μM) in

buffer containing 25 mM HEPES, 100 mM NaCl, 5% glycerol and 0.5 mM EDTA, pH 8.0 on ice for 2 h. Then the ncBAF–NCP complexes were cross-linked with 3 mM bis(sulfosuccinimidyl)suberate (BS3) at room temperature for 30 min. The reactions were quenched with 100 mM Tris, pH 8.0. Sample processing and mass spectrometry data analyses were done as described[13]. The acquired raw data files were searched by the software pLink2 against a protein database containing ncBAF subunits and histones[14]. Precursor mass tolerance was set at 20 parts per million (ppm), and the fragment ion mass tolerance was set to 0.02 Da. The data analysis included spectral processing, potential cross-link detection and peptide pair identification. Results were filtered by an FDR of 5% for cross-linked peptides and visualized by xiVIEW[15]. All the linked site, total spectra, and scores of the XL-MS results are provided (Supplementary Table S2).

Cell culture and lentiviral infection

Human acute myeloid leukemia (AML) cell lines (HL-60, THP-1) and HEK-293T cells were obtained from the American Type Culture Collection (ATCC, Manassas, Virginia, USA). HL-60 and THP-1 cells were cultured in RPMI-1640 medium (Gibco) supplemented with 10% fetal bovine serum (FBS, BioInd) and 1% penicillin-streptomycin. HEK-293T cells were cultured in DMEM medium (Gibco) supplemented with 10% FBS (Gemini) and 1% penicillin-streptomycin. All cells were incubated at 37°C in a humidified atmosphere containing 5% CO_2. The human *BCL7A* coding sequence was cloned into pLVX-IRES-puro vector with a C-terminal

Flag-HA tag to generate the pLVX-WT-BCL7A-puro plasmids. The R11G mutation in BCL7A was introduced by site-directed mutagenesis, resulting in pLVX-mut-BCL7A-puro plasmids. All constructs were verified by Sanger sequencing.

Lentiviral particles were produced by transfecting HEK-293T cells (85% confluency) with packaging plasmids (psPAX2 and pMD2.G) and the transfer vectors, using Neofect transfection reagent (Neofect, TF20121201). Viral supernatants were collected at 48 and 72 h post-transfection, filtered through 0.45-μm sterile filters, and used to infect HL-60 and THP-1 cells in the presence of 8 μg/mL polybrene. After 14 h of incubation, the medium was replaced with fresh complete medium. Puromycin (Solarbio, P8230) selection (1.2μg/mL) was initiated at 48 h post-infection and maintained throughout subsequent culture. Stable expression was confirmed by western blotting.

Cell proliferation assay and Western blot

Stable cells were seeded in 6-well plates (2×10^6 cells/2mL per well) 48 h of puromycin selection. Viable cell numbers were counted every 48 h by 0.4% trypan blue exclusion (Solarbio, C0040) using a hemocytometer. Growth curves were generated by plotting viable cell numbers over time normalized to the vector group, based on three independent biological replicates, each measured in triplicate.

HL-60 and THP-1 cells were counted and 1×10^6 cells were lysed in 100μL SDS loading buffer (50mM Tris-HCL [PH 6.8], 10% glycerol, 2% SDS, 0.1% bromophenol blue, 1% β-mercaptoethanol). The lysates were sonicated (40% amplitude, 6×1 s pulses) and denatured at 95°C for 5 min. Proteins were separated by 15 % SDS-PAGE, transferred to the nitrocellulose membranes, and blocked with 5% bovine serum albumin (BSA) in TBST for 30 min. Membranes were incubated with anti-BCL7A (abcam, #ab259833, 1:1000) and anti-β-TUBULIN (Yeasen, 30302ES60, 1:5000) overnight at 4°C, followed by HRP-conjugated secondary antibodies (Easybio, BE0101, 1:10000) for 1 h at room temperature. Signals were detected using chemiluminescence.

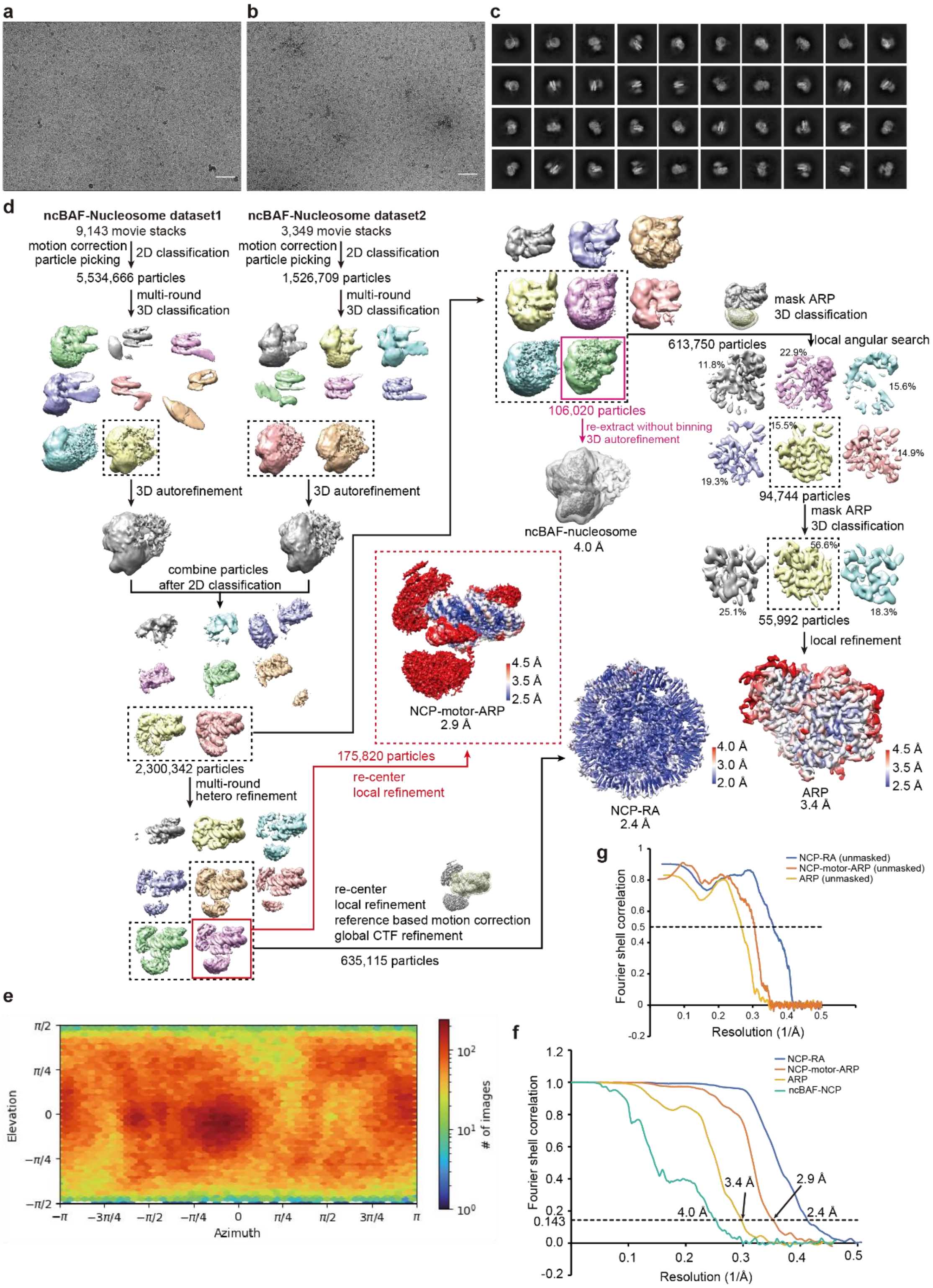

Supplementary Figure 1 | Cryo-EM analysis of the ncBAF-nucleosome complex.

(**a, b**) Representative cryo-EM micrographs of the two datasets. Scale bar: 50 nm. (**c**) 2D class averages of characteristic projection views of cryo-EM particles. (**d**) Flowchart of the cryo-EM data processing. (**e**) Angular distributions of the cryo-EM

particles in the final round of refinement. (**f**) Resolution estimation of the EM maps according to the gold standard Fourier shell correlation (FSC) curves. (**g**) Model-map FSC plot calculated by Phenix between the map and the model.

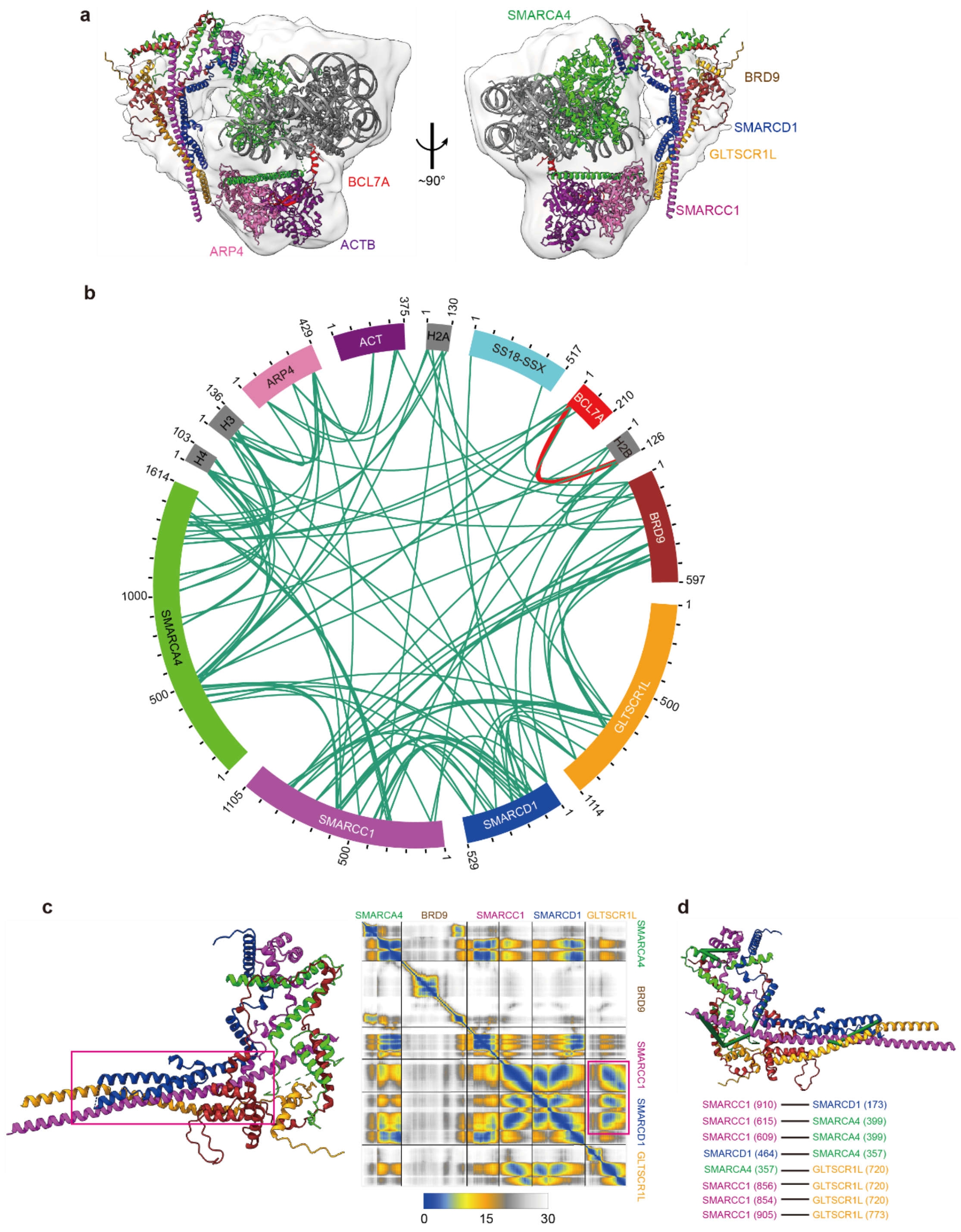

Supplementary Figure 2 | Structural model of the ncBAF-NCP complex and inter-molecular interactions of the ncBAF-nucleosome complex detected by XL-MS.

(a) The predicted model of SRM by Alphafold3 is fit into the overall map of ncBAF-NCP complex. (**b**) The crosslinking-MS results of ncBAF-NCP complex,

supporting the model of SRM module. The crosslinking between BCL7A N-terminal (K15) and H2B (K108) was colored red, as an evidence of RA of BCL7A closed to acidic patch of the nucleosome in solution. (**c**) The Alphafold3 predicted model of SRM module with the PAE values plot. Lower PAE values indicate higher prediction confidence. (**d**) The high-confidence cross-linked sites were mapped onto the predicted model of SRM.

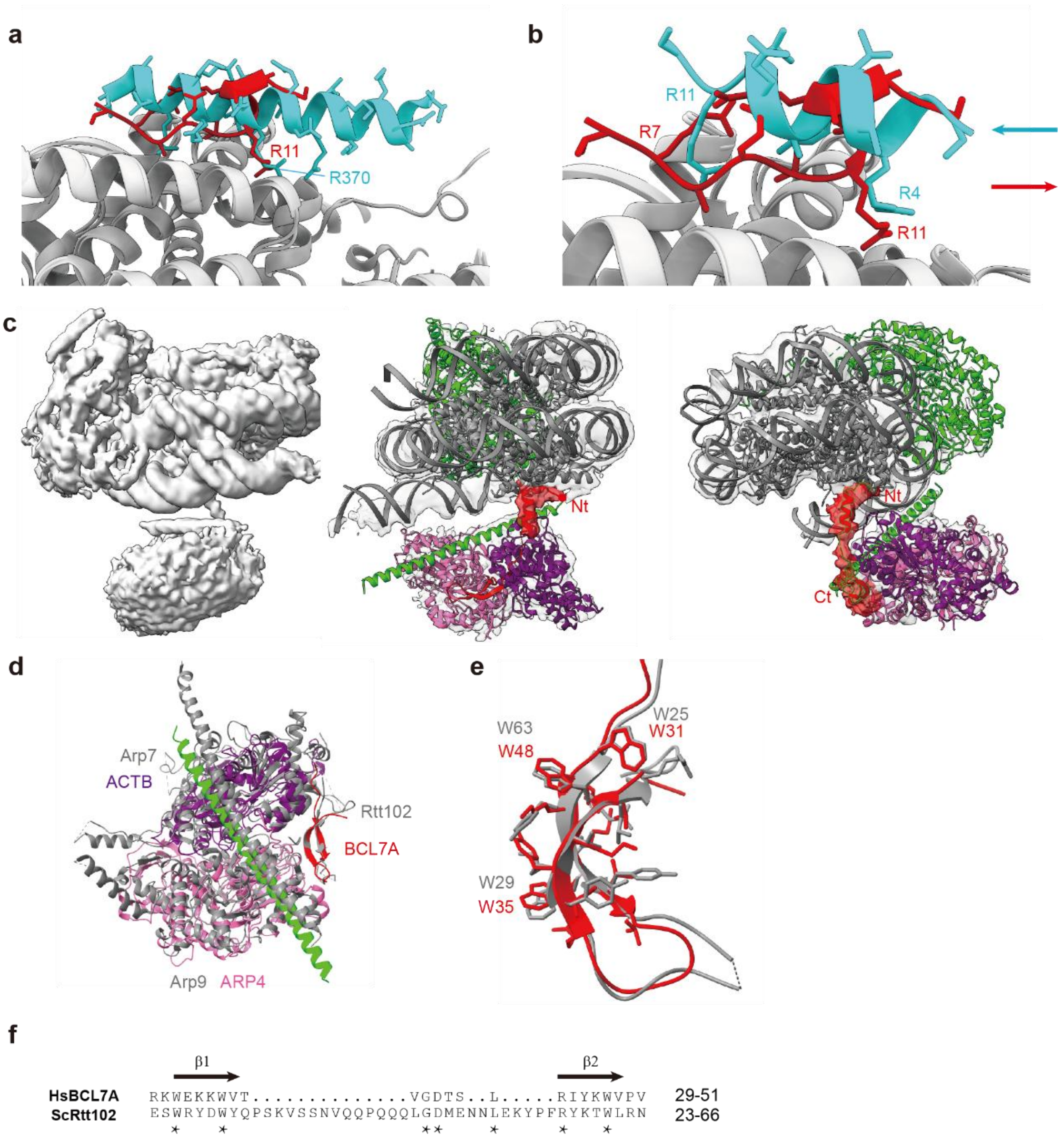

Supplementary Figure 3 | Structural comparison of the ARP module and the local density maps.

(**a**) Structural comparison of the RA of BCL7A and FH of SMARCB1 (PDB code: 6LTJ), showing the canonical arginine anchor R11 of BCL7A and R370 of SMARCB1. (**b**) Structural comparison of the RA region of isolated BCL7A (cyan, PDB code: 9QAJ) and in the context of ncBAF (red) bound to the NCP (grey). The structures of NCP are aligned. (**c**) The structure of NCP-motor-ARP region in ncBAF complex exhibits reasonable continuity, facilitating determination of the structural

trajectory of the RA motif of BCL7A. (**d, e**) Structural comparison of the ARP module of human ncBAF and yeast RSC. The models are aligned according to BCL7A and Rtt102. (**f**) Structure and sequence alignment of BCL7A and Rtt102.

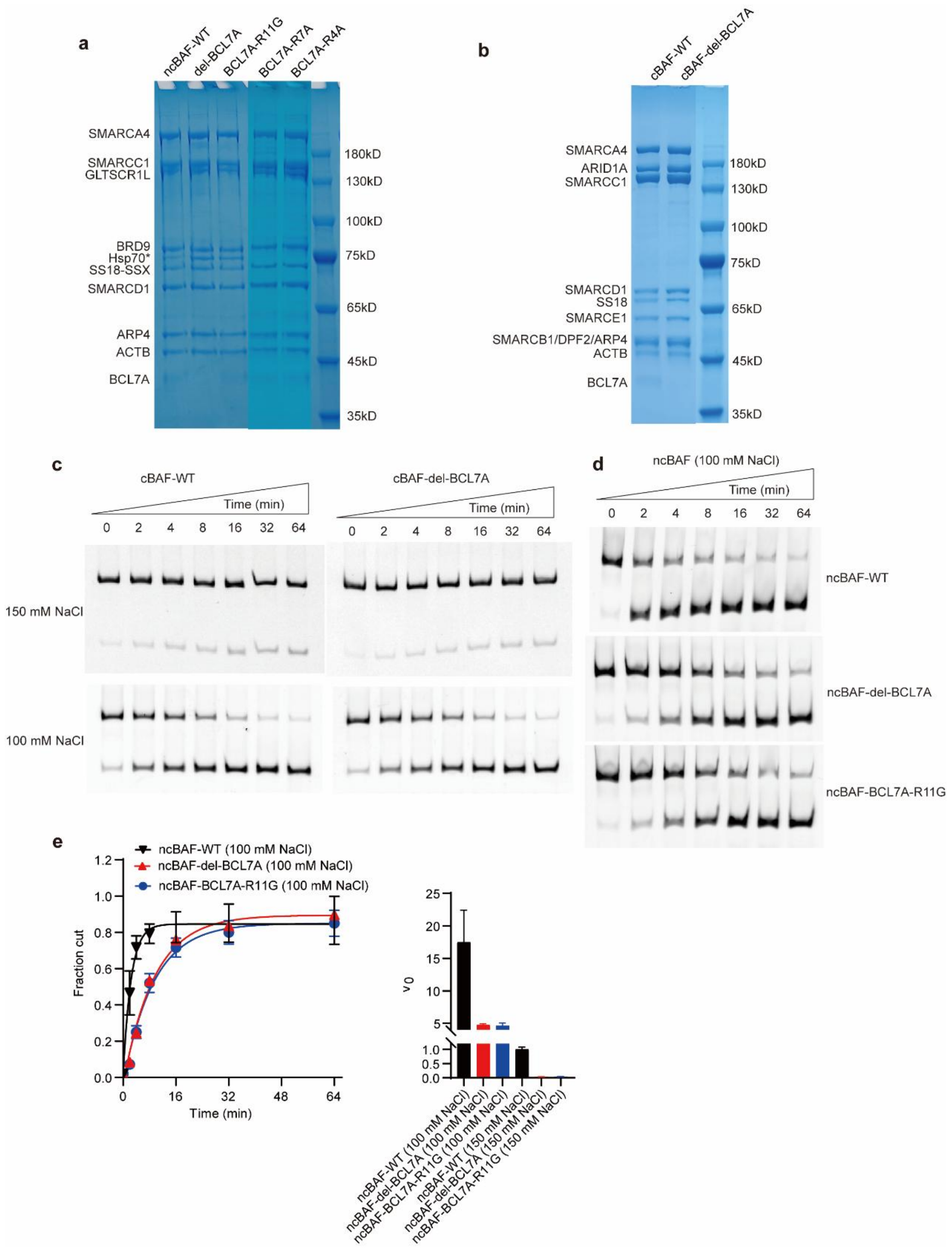

Supplementary Figure 4 | Additional biochemical analyses of the BCL7A mutant complex

(a) Representative SDS-PAGE gels of the WT and mutant ncBAF complexes.

(b) Representative SDS-PAGE gels of the WT and BCL7A deletion cBAF complexes. (c) Representative gels of the chromatin remodeling assays of WT and BCL7A deletion cBAF complexes at 150 mM and 100 mM NaCl. (d) Representative gels of the chromatin remodeling assays of WT and BCL7A deletion or R11G mutant ncBAF complexes at 100 mM NaCl. (e) Quantification of the chromatin remodeling activities of WT (black) and BCL7A deletion (red) or R11G (blue) mutant complexes at 100 mM NaCl. Normalized initial remodeling rates (v_0) are shown on the right. Data are mean ± s.d. (n = 3 technical replicates).

Additional references:

1 He, S. *et al.* Structure of nucleosome-bound human BAF complex. *Science* **367**, 875-881 (2020).

2 Yuan, J., Chen, K., Zhang, W. & Chen, Z. Structure of human chromatin-remodelling PBAF complex bound to a nucleosome. *Nature* **605**, 166-171 (2022).

3 Zheng, S. Q. *et al.* MotionCor2: anisotropic correction of beam-induced motion for improved cryo-electron microscopy. *Nature Methods* **14**, 331-332 (2017).

4 Rohou, A. & Grigorieff, N. CTFFIND4: Fast and accurate defocus estimation from electron micrographs. *Journal of Structural Biology* **192**, 216-221 (2015).

5 Scheres, S. H. W. RELION: Implementation of a Bayesian approach to cryo-EM structure determination. *Journal of Structural Biology* **180**, 519-530 (2012).

6 Punjani, A., Rubinstein, J. L., Fleet, D. J. & Brubaker, M. A. cryoSPARC: algorithms for rapid unsupervised cryo-EM structure determination. *Nature Methods* **14**, 290-296 (2017).

7 Pettersen, E. F. *et al.* UCSF Chimera—A visualization system for exploratory research and analysis. *Journal of Computational Chemistry* **25**, 1605-1612 (2004).

8 Evans, R. *et al.* Protein complex prediction with AlphaFold-Multimer. bioRxiv 2021.10.04.463034 (2022).

9 Abramson, J. *et al.* Accurate structure prediction of biomolecular interactions with AlphaFold 3. *Nature* **630**, 493-500 (2024).

10 Emsley, P. & Cowtan, K. Coot: model-building tools for molecular graphics. *Acta Crystallographica Section D* **60**, 2126-2132 (2004).

11 Afonine, P. V. *et al.* Real-space refinement in PHENIX for cryo-EM and crystallography. *Acta Crystallographica Section D* **74**, 531-544 (2018).

12 Pettersen, E. F. *et al.* UCSF ChimeraX: Structure visualization for researchers, educators, and developers. *Protein Science* **30**, 70-82 (2021).
13 Ye, Y. *et al.* Structure of the RSC complex bound to the nucleosome. *Science* **366**, 838-843 (2019).
14 Chen, Z.-L. *et al.* A high-speed search engine pLink 2 with systematic evaluation for proteome-scale identification of cross-linked peptides. *Nature Communications* **10**, 3404 (2019).
15 Combe, C. W., Graham, M., Kolbowski, L., Fischer, L. & Rappsilber, J. xiVIEW: Visualisation of Crosslinking Mass Spectrometry Data. *Journal of Molecular Biology* **436**, 168656 (2024).

Supplementary Table 1. Cryo-EM data collection, refinement and validation statistics

	#1 ncBAF-NCP (EMD-65852)	#2 NCP-motor-ARP (EMD-65851) (PDB-9WBZ)	#3 NCP-RA (EMD-65853) (PDB-9WC0)	#4 ARP (EMD-65854) (PDB-9WC1)
**Data collection and processing**				
Magnification	81,000×	81,000×	81,000×	81,000×
Voltage (kV)	300	300	300	30
Electron exposure (e–/Å^2)	50	50	50	50
Defocus range (μm)	-1.4— -1.8	-1.4— -1.8	-1.4— -1.8	-1.4— -1.8
Pixel size (Å)	0.54125	0.54125	0.54125	0.54125
Symmetry imposed	C1	C1	C1	C1
Initial particle images (no.)	7,061,375	7,061,375	7,061,375	7,061,375
Final particle images (no.)	106,020	175,820	635,115	55,992
Map resolution (Å)	4.0	2.9	2.4	3.4
FSC threshold	0.143	0.143	0.143	0.143
Map resolution range (Å)		2.5-4.5	2.0-4.0	2.5-4.5
**Refinement**				
Initial model used (PDB code)		7VDT		
Model resolution (Å)		3.2	2.6	3.6
FSC threshold		0.5	0.5	0.5
Model resolution range (Å)		2.8-3.2	2.4-2.6	2.9-3.6
Map sharpening *B* factor (Å^2)		-63.9	-85.8	-96.0
Model composition				
Non-hydrogen atoms		24606	12431	7206
Protein residues		2294	777	916
Ligands		3		
B factors (Å^2)				
Protein		45.96	17.68	68.47
Ligand		451.71		
R.m.s. deviations				
Bond lengths (Å)		0.003	0.004	0.003
Bond angles (°)		0.465	0.603	0.518
EMRinger score		1.64	3.97	3.38
Validation				
MolProbity score		2.00	1.10	1.22
Clashscore		20.99	3.13	4.39
Poor rotamers (%)		0.00	0.00	0.00
Ramachandran plot				
Favored (%)		97.08	98.68	98.35
Allowed (%)		2.92	1.32	1.65
Disallowed (%)		0.00	0.00	0.00
